# Prevalence and risk factors of posterior vitreous detachment in a Chinese adult population: the Handan eye study

**DOI:** 10.1186/1471-2415-13-33

**Published:** 2013-07-16

**Authors:** Zhijun Shen, Xinrong Duan, Fenghua Wang, Ningli Wang, Yi Peng, David TL Liu, Xiaoyan Peng, Sizhen Li, Yuanbo Liang

**Affiliations:** 1 Beijing Tongren Eye Center, Beijing Tongren Hospital, Capital Medical University; Beijing Ophthalmology & Visual Science Key Lab, Beijing, China; 2 Department of Ophthalmology and Visual Sciences, the Chinese University of Hong Kong, Hong Kong, China

**Keywords:** Posterior vitreous detachment, Prevalence, Population-based, Risk factor

## Abstract

**Background:**

To describe the prevalence and associations of posterior vitreous detachment (PVD) in a rural adult Chinese population.

**Methods:**

All eligible subjects were requested to carry out a comprehensive eye examination; PVD was a pre-specified outcome variable and was determined via biomicroscopical examination (slit-lamp biomicroscopy) with a +90-D preset lens after mydriasis. Prevalence was standardized to China population census (2000).

**Results:**

5890 (86.2%) subjects completed the examination of slit-lamp biomicroscopy with a +90-D lens. PVD was present in 160 participants (2.7%); the standardized prevalence was 2.0% (95% confidence interval [CI], 1.6-2.3%). PVD developed increasingly with age (P for trend < 0.001) for both men and women. Using a multivariate regression model, older people were found to run a higher risk of developing PVD than younger people, and women were found to have a higher risk than men (OR, 2.9; 95% CI, 1.5-5.9). Diabetes, hypertension, smoking, drinking, and intraocular pressure (IOP) were not significantly associated with PVD.

**Conclusions:**

About one in fifty people is found to have PVD in this population-based study. Age and female are independently associated with PVD occurrence.

## Background

The vitreous body plays important roles in both intraocular metabolic exchange and the stability of the retina. Losing its gel-like consistency with aging, the vitreous body may eventually detach from the retina and optic disc, clinically described as posterior detachment of the vitreous body (PVD) [1]. When PVD occurs, peripapillary ring and posterior hyaloid membranes are seen. PVD is related to the pathogenesis of retinal tears, subsequent retinal detachment, and some other ocular diseases such as macular holes and vitreous or retinal hemorrhages [2-5]. The incidence of complications in symptomatic PVD is as high as 24.1% [6,7].

However, information on the prevalence of PVD is largely lacking. The limited data to date have mostly come from hospital-based and post-mortem studies [1,3,8-10]. Age [11], gender [12], myopia [3,8], retinitis pigmentosa [13], and ethnic background [4] etc. have been studied with respect to the development of PVD. In this report, we described the prevalence and associations of PVD in a large sample of Chinese population aged 30+ years, living in Handan, Hebei Province, China.

## Methods

### Study design and population

The Handan Eye Study is a population-based study designed to determine the prevalence of blindness, visual impairment, and common eye diseases in a rural population of northern China. Details of the study design, sampling plan, and baseline data are reported elsewhere [14-16]. Ethics committee approval was obtained from Beijing Tongren Hospital Ethical Committee and all participants signed informed consent before the study.

In brief, a total of 6830 Han Chinese aged 30 years and older were randomly selected from 13 villages in Yongnian County, Handan City and were invited to participate in the Handan Eye Study between October 2006 and October 2007. 80% of the population work as peasants, and 98% are Hans. Socioeconomically, they are at the average level in mainland China [17].

All eligible individuals underwent a comprehensive eye examination after pupil dilation, including standardized refraction and biomicroscopical examination (slit-lamp biomicroscopy) with a +90-D preset lens by a trained ophthalmologist.

### Examination and definitions

In this study, a senior retina specialist (DXR) would diagnose the vitreous conditions by observing kinetic movement of the posterior vitreous during saccadic movement of the eyes via a slit-lamp biomicroscopy with a +90-D preset lens. PVD was defined as separation of the posterior vitreous cortex from the retinal surface with a peripapillary ring and/or an optically empty subhyaloid space [18,19]. The dynamic vitreous examination was used to decrease the rate of misdiagnosis of PVD [18,20,21].

Refractive error was defined as spherical equivalent (sphere + cylinder/2) [15]. High myopia was defined as spherical equivalent (SE) < -6.0D, moderate myopia was SE ≥ -6.0D and ≤ -1.0D, and mild myopia and hypermetropia were combined as SE > -1.0D.

Diabetes mellitus was diagnosed from self-reported history of medication and/or a fasting plasma glucose ≥7.0 mM (126 mg/dL). Hypertension was defined as having a history of hypertension on medication or elevated blood pressure when measured in the examination (diastolic blood pressure ≥90 mmHg or systolic blood pressure ≥140 mmHg). Smoking referred to 1 to 100 cigarettes per day and continued to 100 cigarettes. Drinking referred to often drinking (at least one time per week at least 50 g per time).

### Statistical analysis

Statistical analysis was performed by using a statistical software package (SAS9.1.3). All statistical tests were significant if P values were less than 0.05. The association with PVD, such as gender, intraocular hypertension and health was estimated by the odds ratio (OR) and its 95% confidence interval (95% CI) with multivariate logistic regression model.

## Results

Of the 6830 participants, 5890 (86.2%) completed the examination of slit-lamp biomicroscopy with a +90-D preset lens in the county hospital. 807 (11.8%) at temporary study sites in the villages and 114 (1.7%) at home completed the preset lens examination without mydriasis, which might have made it impossible to detect PVD, and, therefore, we excluded these participants from the calculation of prevalence. An additional 19 (0.28%) people had no PVD data because of media opacities or missing data. The mean age of the participants included in the analysis was 52.0 ± 11.5 years and 54.8% were female. Those who did not complete the examination were older, having higher income, with less education, and male gender and were more likely to have no health insurance.

### Prevalence of PVD

PVD was present in 160 participants (2.7%). Standardized directly by age and gender according to the national population census (China 2000), the prevalence of PVD was 2.0% (95% CI, 1.6-2.3). PVD was present only in 3 participants who were 30+ years old and 40+ years old. PVD was detected in 53 (53/2660 = 1.99%) male subjects and 107 (107/3230 = 3.31%) in female subjects. PVD developed increasingly with age (P for trend < 0.001) for both men and women (Table 1). Women were more likely to have PVD (3.31% vs.1.99%), and there was a significant difference between man and women after age adjustment (P < 0.001).

**Table 1:** Prevalence of posterior vitreous detachment in the Handan Eye Study

**Age group**	**Males**		**Females**		**Total**	
	**N**	**% (95% CI**^**a**^**)**	**N**	**% (95% CI**^**a**^**)**	**N**	**% (95% CI**^**a**^**)**
30~39	426	0.47 (0,1.12)	592	0.17 (0,0.5)	1018	0.29 (0,0.62)
40~49	538	0.19 (0,0.56)	661	0.3 (0,0.72)	1199	0.25 (0,0.53)
50~59	998	0.8 (0.25,1.35)	1233	2.03 (1.24,2.82)	2231	1.48 (0.98,1.98)
60~69	488	4.92 (3,6.84)	500	7.8 (5.45,10.15)	988	6.38 (4.86,7.9)
70+	210	8.57 (4.78,12.36)	244	16.39 (11.75,21.03)	454	12.78 (9.71,15.85)
total	2660	1.99 (1.46,2.52)	3230	3.31 (2.7,3.93)	5890	2.72 (2.3,3.13)
P for trends	<0.001		<0.001		<0.001	

a: confidence interval.

### PVD and refractive error

Of the 5890 subjects, refractive error was found in 5861 in at least one eye. The prevalence of PVD was higher in subjects with high myopia [> -6.0 diopters (D)] (6.6%) than in subjects with moderate myopia (<= -1.0D and> = -6.0D, p = 0.013) (2.3%) and those with mild myopia and hyperopia (>-1.0D, p = 0.01) (2.6%). However, the trend of increasing prevalence with myopic SE was higher only in 30~39-year-old group (P for trend = 0.005), but not in other age groups (P > 0.05). The prevalence of PVD even decreased with the increase of myopic SE in 70 + -year-old group (P for trend =0.023). In high myopia group, age was not associated with prevalence of PVD (p for trend = 0.45), while in mild and moderate groups and hyperopia group, prevalence of PVD increased remarkably with age (p for trend < 0.001). (Table 2)

**Table 2:** Prevalence of posterior vitreous detachment by refractive error and age in the Handan eye study

**Age group**	**SE > -1D**		**SE ≥ -6D and ≤ -1D**		**SE < -6D**		**P for trend (SE**^**b**^**)**
	**N**	**% (95% CI**^**a**^**)**	**N**	**% (95% CI**^**a**^**)**	**N**	**% (95% CI**^**a**^**)**	
30~39	764	0.13 (0,0.39)	234	0.43 (0,1.27)	11	9.09 (0,26.08)	0.005
40~49	1046	0.19 (0,0.45)	134	0.75 (0,2.21)	13	0	0.352
50~59	1998	1.4 (0.88,1.92)	191	1.05 (0,2.5)	39	5.13 (0,12.05)	0.311
60~69	850	6.35 (4.71,7.99)	109	5.5 (1.22,9.78)	25	8 (0,18.63)	0.996
70+	298	15.44 (11.34,19.54)	131	6.11 (2.01,10.21)	18	11.11 (0,25.63)	0.023
total	4956	2.64 (2.2,3.09)	799	2.25 (1.22,3.28)	106	6.6 (1.88,11.33)	0.329
P for trend (age)	<0.001		<0.001		0.45		

a: confidence interval; b: Spherical equivalence.

### PVD and associations

Table 3 showed the factors associated with PVD using multivariate logistic regression models in the Handan Eye Study. Female (OR, 2.92; 95% CI, 1.45-5.86) and age were associated with PVD in persons ≥ 50 years of age. The odd of PVD in those aged ≥ 70 years was 62.7 (95% CI, 14.56-269.88) times the odds for those aged 30 to 39 years. Diabetes, hypertension, drinking, smoking, IOP, and refractive error were not found to be associated with PVD in multivariate logistic regression models.

**Table 3:** Factors associated with posterior vitreous detachment using multivariate logistic regression models in the Handan eye study

**Factors**	**OR**^**b**^**(95% CI**^**a**^**)**	**P**
Hypertension	0.97(0.65-1.47)	0.903
age 40~49 vs. 30~39	1.19(0.20-7.16)	0.849
age 50~59 vs. 30~39	6.65(1.57-28.22)	0.010
age 60~69 vs. 30~39	28.30(6.72-119.19)	<0.001
age 70+ vs. 30~39	62.68(14.56-269.88)	<0.001
Female vs. male	2.92(1.45-5.86)	0.003
Diabetes	0.48(0.22-1.05)	0.066
Drinking		
current vs. never	0.74(0.34-1.63)	0.455
past vs. never	1.90(0.79-4.58)	0.152
Smoking		
current vs. never	1.80(0.81-3.98)	0.146
past vs. never	1.12(0.35-3.62)	0.851
SE^c^		
SE ≥ -6D and ≤ -1D vs. SE > -1D	0.60(0.33-1.10)	0.100
SE < -6D vs. SE > -1D	1.62(0.61-4.34)	0.355
IOP^d^(per 1 mmHg)	0.98(0.92-1.04)	0.526

a: confidence interval; b: odds ratio; c: spherical equivalence; d: intraocular pressure.

## Discussion

This study studies the prevalence of PVD, its relationship with age, gender, and refractive error from a population-based cross-sectional study of Chinese adults aged 30 years and more. We report an overall prevalence of 2.7% for PVD, and 6.6% in high myopia. This study shows female, older age, and young people with high myopia were risk factors for PVD.

Müller [22] described PVD histopathologically as early as 1856. In 1972, Foos reported that 24.5% had PVD in one (7%) or both (17%) eyes in a large post-mortem study of 786 (1572 eyes) adult subjects > =20 years old [9]. Hayreh examined 1481 subjects (2962 eyes) including all consecutive patients seen in an Ocular Vascular Clinic over an 8-year period and found that PVD was seen in 37% (bilateral in 27.5% and unilateral in 9.7%) of the patients [1]. Gella reported that the prevalence of PVD in subjects with type II diabetes mellitus was 63.3% [23]. In our study, PVD was present in 12.78% in senior people aged 70 years and over. The prevalence among those aged 70 and above was still lower than reports from others studies [1,9,10]. These studies, on one hand, were mostly clinic-based and the subjects attending an eye clinic were more likely to have vision symptoms such as floaters, and thus could have led to a higher prevalence. On the other hand, in this study we diagnosed PVD based solely on examiner’s clinical impression without B-scan ultrasonography or optic coherence tomography (OCT) which could detect PVD more easily [24]; Besides, the fact that the low prevalence of myopia in studied rural population was compared with white population (9.5% versus 19.0% for SE < -1.0D) might be an alternative explanation [15,25]. The rural and low literacy population in this study may be a reason for the low rate of myopia and PVD.

The increasing rate of PVD with age was consistent with previous reports [1,4,10,12,23]. In the post-mortem study, Foos found PVD in one or both eyes in 0.4% of subjects aged 20–49 years, in 7.2% of subjects aged 50–59 years, in 22% of subjects aged 60–69 years and in 60% of subjects aged 70 or more [9]. Hayreh found the frequency increased from 4.7% in subjects younger than 45 years to 20.4% in subjects aged 45–65 years, and to 58.4% in subjects older than 65 years [1]. With kinetic B-mode sonography, Weber-Krause and Eckardt found PVD in 29% of persons 65 to 69 years old and in 57% of those between 80 to 89 years old [10]. In our study, the frequency of PVD increased from 0.47% in subjects younger than 40 to 8.57% in subjects older than 70 for males, and from 0.17% to 16.39% for females. Age was independently associated with PVD in persons ≥50 years of age.

Gender was another independent factor in the process of PVD. The risk of PVD in females was nearly two times as higher as that in males (OR = 2.9, 95% CI 1.45-5.86). Foos [9] and Hayreh [1] also found that PVD was significantly more frequent in women than in men, and was even affected by other factors such as age and refractive error. The reason is not clear. The relatively reduced hyaluronic acid concentrations in female eyes described by Larsson might explain the differences in the prevalence of PVD between females and males [26]. Chuo et al. documented a strong and consistent association between PVD and a history of menopause. It is possible that the vitreous collagen or the vitreoretinal interface may be influenced by perimenopausal hormonal changes [12].

In our study, those with high myopia had higher prevalence (6.6%) compared with normal people (2.7%). High myopia seems to increase the risk of PVD in younger people but not in elderly and high myopia was not found to be associated with PVD in multivariate logistic regression models. This was inconsistent with previous hospital-based studies [3,8,12]. The lower rate (1.8%) of high myopia in the population may decrease the power to detect this association statistically. Nevertheless, Berman and Michaelson found that there was no difference in total amount of hyaluronic acid and collagen between highly myopic and emmetropic eyes, and no age-related increase in total amount of hyaluronic acid and collagen was observed in face of increase in the total volume of vitreous [27].

**Figure 1:**
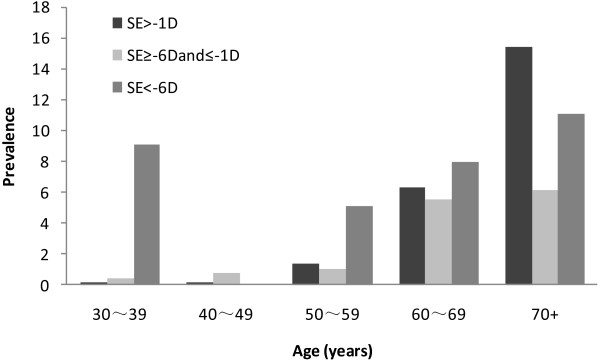
**Prevalence of posterior vitreous detachment by refractive error and age in the Handan Eye Study. **The age of onset of PVD might be earlier in eyes with high myopia than in lower myopic eyes or normal eyes.

Though high myopia was not observed to be associated with the prevalence of PVD in this study, the age of onset of PVD might be earlier in eyes with high myopia than in lower myopic eyes or normal eyes (Figure 1). And in young group of 30–39 years old, myopia was a risk factor for PVD (P for trend = 0.005). This was consistent with previous reports [3,8,28]. Rieger first investigated the prevalence of PVD in high myopia and found that PVD developed increasingly with age and higher degrees of myopia [28]. Hiroyuki Morita et al. investigated 329 eyes with high myopia of more than -8.25D and found PVD was present in 157(47.8%) eyes. He found that PVD had already developed in some of the patients with highly myopic vision by 20 to 29 years of age [3]. In our study, prevalence of PVD in those 30–39 years old with high myopia was 9.1% and 6.0% among persons aged 59 years old or younger. In contrast, 0.7% were observed with PVD among moderate myopia group, and 0.8% among mild myopia or hyperopia group (SE > -1D).

No association was found between PVD and diabetics, hypertension, smoking, drinking or IOP. Foos et al., after comparing the prevalence of PVD in their autopsy study between diabetic subjects and the general population, found that PVD was significantly more common in the eyes of diabetics including those with no retinopathy [29]. However, Hayreh et al. found that diabetic patients did not differ significantly in the frequency of PVD from non-diabetic subjects [1]. Chuo et al. found no association between PVD and smoking, drinking, and hypertension [12]. Hayreh et al. reported that no significant association between frequency of PVD and open-angle glaucoma [1]. More researches are needed to confirm these results.

Our study has limitations. Although PVD was a pre-specified outcome variable in this study and based on a large population and retinal examination was conducted by an experienced ophthalmologist. We may have underestimated the prevalence: 1) The diagnosis of PVD was examiner-dependent without objective verifications like B scan or OCT examinations, although a prospective study showed that ultrasonography and slit-lamp biomicroscopy were the most reliable noninvasive clinical techniques to detect PVD prior to vitrectomy [19]. In this study, Triamcinoloneacetonide was used for chromodissection to visualize the posterior vitreous cortex with little or no risk of retinal toxicity. Ultrasonography and slit-lamp biomicroscopy had the highest rate of correct preoperative assessments, which was 83% and 76%, respectively. 2) We did not differentiate between partial and complete PVD which might carry pathogenesis meanings. 3) A mixed bias might have existed. The examined participants were more females than males, which could result in an overestimate of the prevalence, and the lower ratio of people aged 60 years and over could lead to an underestimate.

## Conclusions

In conclusion, about one in fifty people were found to have PVD in this adult Chinese population-based study. PVD, as a pre-specified outcome variable, was diagnosed on the basis of slit-lamp biomicroscopy with a +90-D preset lens after mydriasis alone without OCT or B-scan. Age and female were independently associated with PVD occurrence, while diabetics, hypertension, smoking, drinking, and IOP were not associated with PVD. High myopia was found to increase the risk of PVD in younger people but not in elderly.

## Competing interests

The authors declare that they have no competing interests.

## Authors’ contributions

NLW participated in the design of the study, drafting the article, obtaining funding and supervision. ZJS, YBL participated in providing conception and design, drafting the article, data analysis and interpretation. XRD, FHW, SZL carried out the eye examination and participated in data acquisition, providing conception and design. YP, DTL participated in data analysis, revising the article. XYP participated in providing conception and design, data interpretation, revising the article. All authors read and approved the final manuscript.

## Pre-publication history

The pre-publication history for this paper can be accessed here:

http://www.biomedcentral.com/1471-2415/13/33/prepub
